# Genetic Insights Into the Link Between Restless Legs Syndrome and Diabetic Nephropathy Risk

**DOI:** 10.1002/brb3.70696

**Published:** 2025-07-21

**Authors:** Yun Lin, Xiaorui Cai, Haohao Chen, Xiaoling Tang

**Affiliations:** ^1^ Department of Pharmacy Shantou Central Hospital Shantou Guangdong Province China; ^2^ Department of Pharmacy The Affiliated Cancer Hospital of Shantou University Medical College Shantou Guangdong Province China; ^3^ Department of Pharmacy The First Affiliated Hospital of Shantou University Medical College Shantou Guangdong Province China; ^4^ Department of Nephrology Shantou Central Hospital Shantou Guangdong Province China

**Keywords:** causal inference, diabetes mellitus, diabetic nephropathy, Mendelian randomization, restless legs syndrome

## Abstract

**Background:**

Restless legs syndrome (RLS) is a common neuro‐sensory disorder associated with various metabolic diseases, including diabetes mellitus and its complications. Observational studies suggest a potential association between RLS and diabetic complications; however, the causal relationship remains unclear due to confounding factors and reverse causation. This study aims to assess the causal relationship between RLS and diabetes, including its complications, using a bidirectional Mendelian randomization (MR) approach.

**Methods:**

Genetic instruments derived from the latest genome‐wide association study (GWAS) data for RLS, Type 1 diabetes, Type 2 diabetes, and diabetic complications (diabetic nephropathy, diabetic retinopathy, and diabetic neuropathy) were selected on the basis of MR assumptions. For causal inference, RLS was used as the exposure, whereas diabetes and its complications were considered outcomes. Reverse MR analyses were performed to assess potential causal effects of diabetes and its complications on RLS. Primary analysis used the inverse‐variance weighted (IVW) method, with IVW radial and robust adjusted profile score (RAPS) as supplementary methods. Heterogeneity, pleiotropy, and robustness were assessed in both discovery (UK Biobank) and validation (FinnGen) datasets.

**Results:**

Forward MR analysis revealed a significant causal effect of RLS on the risk of diabetic nephropathy in both the discovery (IVW: OR = 1.049, *p* = 0.0238) and validation cohorts (IVW: OR = 1.067, *p* = 0.0028). However, no significant causal relationships were found for other primary outcomes, including Type 2 diabetes (IVW: OR = 1.011, 95% CI: 0.994–1.029) and Type 1 diabetes (IVW: OR = 0.995, 95% CI: 0.967–1.023). Sensitivity analyses showed no evidence of heterogeneity or horizontal pleiotropy. Reverse MR analysis did not demonstrate a causal effect of diabetes or its complications on RLS.

**Conclusions:**

The findings suggest that RLS causally increases the risk of diabetic nephropathy. Early recognition and management of RLS in patients with diabetes may help prevent or delay the progression of nephropathy. Further studies are warranted to explore underlying mechanisms and potential clinical interventions.

## Graphical abstract

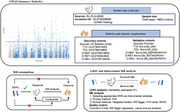

## 1 Introduction

Restless legs syndrome (RLS) is a common neuro‐sensory disorder characterized by an uncontrollable urge to move the legs, which is often accompanied by discomfort and worsens during periods of rest (Trenkwalder et al. 2018). The global prevalence of RLS is estimated to be 7.12% among adults, affecting approximately 356 million individuals (Song et al. 2024). RLS is associated with several systemic diseases, including cardiovascular conditions, hypertension, migraine, and Parkinson's disease. This association is particularly pronounced in diabetes, where RLS may be exacerbated by increased sympathetic activity and metabolic dysregulation (Manconi et al. 2021; Trenkwalder et al. 2016).

Observational studies have indicated a potential association between RLS and diabetes, as well as diabetic complications, with some reports showing a higher prevalence of RLS among individuals with diabetes and its complications (Kalra and Gupta 2018; Ning et al. 2022; Cho et al. 2013). However, the causal nature of this relationship remains unclear due to limitations inherent in observational research, such as confounding factors and the possibility of reverse causation. Determining whether RLS contributes to the development of diabetes and its complications, or vice versa, is crucial for developing targeted interventions and improving patient outcomes.

Mendelian randomization (MR) offers a methodological approach to assess causal relationships using genetic variants as instrumental variables (IVs). By leveraging the random allocation of genes at conception, MR minimizes confounding and reduces the risk of reverse causation inherent in observational studies. This approach can provide more robust evidence for causal inferences between exposures and outcomes (Smith and Ebrahim 2003; Boehm and Zhou 2022).

We employed a bidirectional two‐sample MR analysis to investigate the causal relationships between RLS and diabetes, including its complications (nephropathy, retinopathy, and neuropathy). Using published genome‐wide association study (GWAS) data on RLS (Schormair et al. 2024), diabetes, and its complications from the UK Biobank and FinnGen cohorts (Zorina‐Lichtenwalter et al. 2023; Jiang et al. 2021; Sakaue et al. 2021; Chiou et al. 2021), we aimed to determine whether genetic susceptibility to RLS increases the risk of diabetes and its complications, and conversely, whether genetic predisposition to diabetes and its complications influences the risk of RLS. Clarifying these relationships could significantly impact clinical practice, providing insights into the prevention and management of diabetic complications in patients with RLS.

## 2 Methods

### 2.1 Study Overview

**FIGURE 1:**
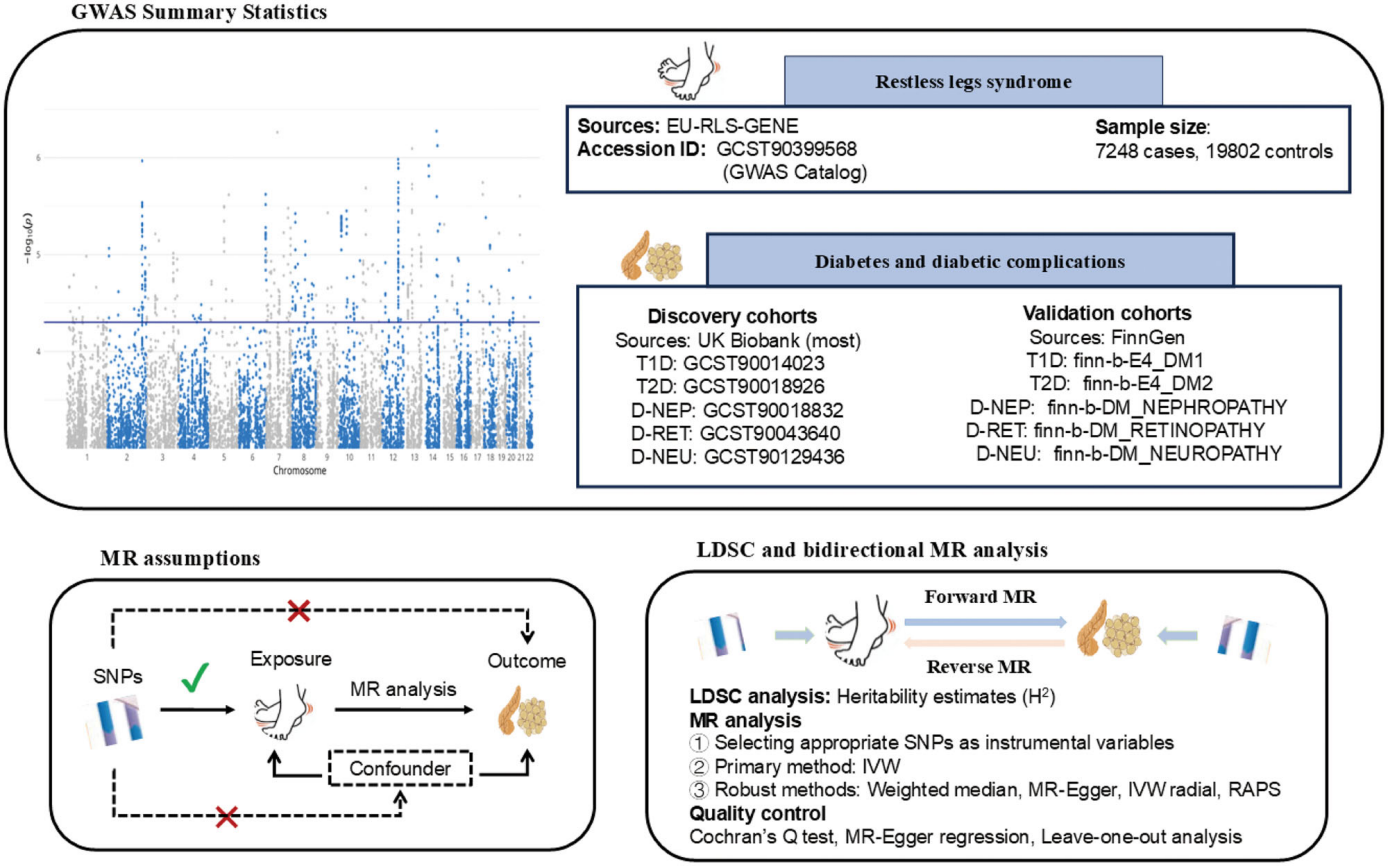
Schematic diagram of the study. D‐NEP, diabetic nephropathy; D‐NEU, diabetic neuropathy; D‐RET, diabetic retinopathy; GWAS, genome‐wide association study; IVW, inverse‐variance weighted; LDSC, linkage disequilibrium score regression; MR, Mendelian randomization; RAPS, robust adjusted profile score; SNPs, single‐nucleotide polymorphisms; T1D, Type 1 diabetes; T2D, Type 2 diabetes.

This study estimated heritability and genetic correlations of the phenotype by analyzing GWAS data. Subsequently, it identified disease‐associated genetic instruments and assessed the bidirectional causal relationship between RLS and diabetes, including its complications, using a two‐sample MR approach. The analysis employed the latest GWAS data for RLS, as well as for Type 1 and Type 2 diabetes (T2D), incorporating additional GWAS data for diabetes‐related complications, such as diabetic nephropathy, retinopathy, and neuropathy. The discovery phase utilized data, primarily from the UK Biobank cohort, whereas validation was performed with data from a FinnGen cohort. In the forward MR analysis, the causal impact of RLS on diabetes and its complications was evaluated across five phenotypes. In contrast, the reverse MR analysis used diabetes and its complications as exposure variables to assess their potential genetic causal effects on RLS. Heterogeneity and pleiotropy analyses were conducted for quality control to ensure the robustness and reliability of the findings. The methodological workflow, encompassing GWAS summary statistics, MR assumptions, linkage disequilibrium score regression (LDSC), and bidirectional MR analysis, is illustrated in Figure 1, whereas a detailed, step‐by‐step flowchart of the entire analytical strategy is provided in Figure S1.

### 2.2 Data Source

RLS GWAS data were obtained from the international EU‐RLS‐GENE consortium, encompassing a discovery meta‐analysis of 7248 cases (2479 males and 4769 females) and 19,802 controls (10,422 males and 9380 females). RLS cases were recruited from multiple European countries, along with Canada (Quebec) and the United States. Diagnoses were conducted by expert neurologists or sleep specialists through face‐to‐face interviews, based on the diagnostic criteria of the International Restless Legs Syndrome Study Group (IRLSSG). This dataset is accessible from the HGRI‐EBI Catalog GWAS under ID GCST90399568 (https://www.ebi.ac.uk/gwas/search?query=GCST90399568), with additional details available in Table 1. For comprehensive information on trait definitions and covariate adjustments, please refer to the original studies (Schormair et al. 2024).

**TABLE 1:** Detailed characteristics of genome‐wide association study (GWAS) datasets used in Mendelian randomization analysis.

ID	Trait	First author	Sex	Population	Sample size (cases/controls)	PMID	Year
GCST90399568	Restless legs syndrome	Schormair B	M and F	European	7248/19,802	36180795	2022
GCST90014023	Type 1 diabetes	Chiou J	M and F	European	18,942/501,638	34012112	2021
GCST90018926	Type 2 diabetes	Sakaue S	M and F	European	38,841/451,248	34594039	2021
GCST90018832	Diabetic nephropathy	Sakaue S	M and F	European	1032/451,248	34594039	2021
GCST90043640	Diabetic retinopathy	Jiang L	M and F	European	308/456,040	34737426	2021
GCST90129436	Diabetic neuropathy	Zorina‐Lichtenwalter K	M and F	European	772/435,199	37219871	2023
finn‐b‐E4_DM1	Type 1 diabetes	NA	M and F	European	5928/183,185	NA	2021
finn‐b‐E4_DM2	Type 2 diabetes	NA	M and F	European	32,469/183,185	NA	2021
finn‐b‐DM_NEPHROPATHY	Diabetic nephropathy	NA	M and F	European	3283/210,463	NA	2021
finn‐b‐DM_RETINOPATHY	Diabetic retinopathy	NA	M and F	European	14,584/202,082	NA	2021
finn‐b‐DM_NEUROPATHY	Diabetic neuropathy	NA	M and F	European	1415/162,201	NA	2021

For the discovery study, Type 1 diabetes (T1D) dataset (ID: GCST90014023) comprises 18,942 patients and 501,638 controls, whereas T2D dataset (ID: GCST90018926) includes 38,841 patients and 451,248 controls. The diabetic nephropathy (D‐NEP) dataset (ID: GCST90018832) contains 1032 patients and 451,248 controls, whereas the diabetic retinopathy (D‐RET) dataset (ID: GCST90043640) includes 308 patients and 456,040 controls. The diabetic neuropathy (D‐NEU) dataset (ID: GCST90129436) consists of 772 patients and 435,199 controls, all of European ancestry. All datasets are available in the HGRI‐EBI GWAS Catalog (https://www.ebi.ac.uk/gwas/home). Validation study GWAS data were obtained from the FinnGen database; further details can be found in Table 1.

### 2.3 Instrument Selection

MR relies on assumptions of correlation, independence, and exclusion. The correlation assumption requires that genetic variants, specifically single nucleotide polymorphisms (SNPs) linked to a particular phenotype, be selected as IVs under stringent criteria. For both forward and reverse MR analyses, IVs for RLS, diabetes mellitus, and diabetic complications were chosen using a significance threshold of *p* < 5E − 8. This widely accepted threshold represents a Bonferroni correction for the approximately one million independent common variants in genomes of European ancestry, effectively controlling the family‐wise error rate at 5% (Uffelmann et al. 2021; Chen et al. 2021). The independence assumption was met by applying a low linkage disequilibrium (LD) threshold (10,000 kb, *r* ^2^ = 0.001). To ensure adequate instrument strength, an *F* value was calculated for each SNP to confirm it exceeded 10, following the approach of Pierce and Burgess (2013). Sensitivity analyses were conducted to verify that causal inference was unaffected by pleiotropy, thereby meeting the exclusion assumption.

### 2.4 Statistical Analysis

LDSC, implemented via the ldscR R package, was employed to investigate genetic associations and interactions among RLS GWAS, diabetic phenotypes, and SNPs. LDSC quantifies polygenic signals and mitigates bias from population stratification, enabling unbiased genetic correlation assessment even with overlapping samples (Bulik‐Sullivan et al. 2015). Furthermore, LDSC was also used to estimate associations between GWAS phenotypes and SNPs. The method also assesses LD analysis to estimate heritability (*h* ^2^), with a significance threshold of *p* < 0.05 indicating significant genetic correlations.

MR analysis was conducted utilizing the TwoSampleMR software package. Within the framework of bidirectional MR analysis, the causal relationship between the exposure and outcome was estimated using the inverse‐variance weighted (IVW) or Wald ratio method. Additionally, we employed the IVW radial approach and the robust adjusted profile score (RAPS) method for causal effect estimation. The IVW method applied a *p* value threshold of <0.05 as the main threshold for evaluating causal effects, without applying Bonferroni adjustments to maintain the exploratory nature of the study. The robustness of MR findings was established if at least one of the alternative methods yielded a similar significant result.

### 2.5 Sensitivity Analyses

A comprehensive suite of sensitivity analyses was conducted to test for potential violations of MR assumptions, particularly heterogeneity and horizontal pleiotropy. Our multi‐pronged strategy included the following: First, we used the MR‐Egger intercept test to specifically detect directional pleiotropy, with an intercept *p* value < 0.05 indicating its presence. Second, we applied Cochran's *Q*‐test to assess heterogeneity among the IVs, as significant heterogeneity can indicate underlying pleiotropy. Third, to further scrutinize and mitigate the influence of individual variants, we employed radial MR methods (implemented via the RadialMR R package) to identify and remove outlying SNPs that might have pleiotropic effects. Finally, a leave‐one‐out sensitivity analysis was performed to ensure that the overall causal estimate was not driven by any single, disproportionately influential SNP (Greco et al. 2015; Burgess and Thompson 2017).

### 2.6 Ethical Approval

Regarding the ethical approvals and informed consent for our study, I would like to clarify that the analysis conducted in this manuscript is based on publicly available GWAS data that had already received the necessary ethical approvals and consent at the time of the original data collection and publication. As no new data collection or direct interaction with participants was performed, additional Institutional Review Board (IRB) approval or informed consent is not required for this analysis.

## 3 Results

### 3.1 Heritability Estimation

**FIGURE 2:**
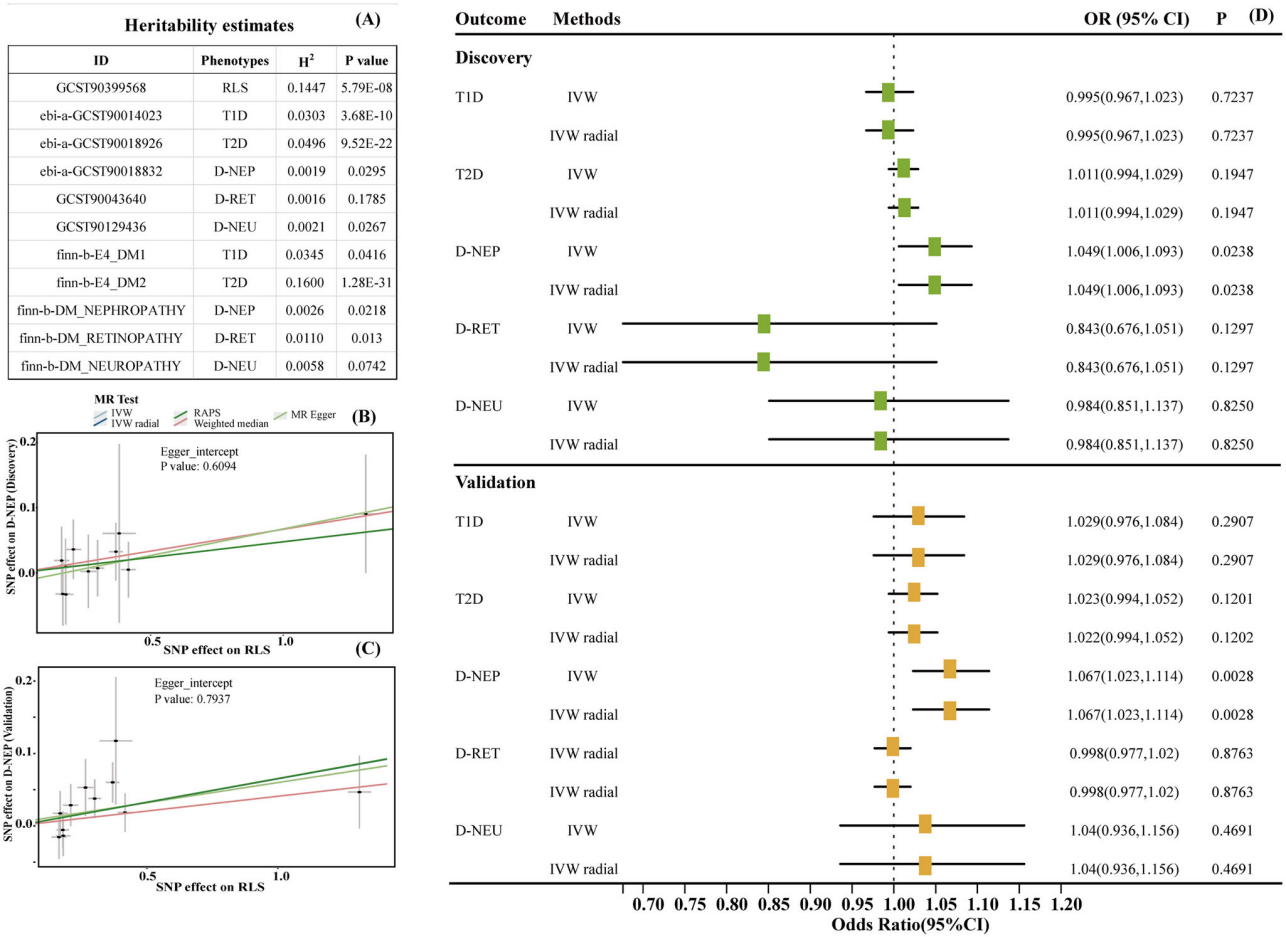
Heritability assessment of GWAS used in the study and forward MR causality evaluation of RLS on diabetes and diabetic complications. (A) Heritability estimates (*h* ^2^) from the heritability assessment; (B) scatter plot showing individual variant regression coefficients in the MR analysis of RLS on D‐NEP during the discovery phase; (C) scatter plot showing individual variant regression coefficients in the MR analysis of RLS on D‐NEP during the validation phase; (D) forest plot presenting causal effect estimates of RLS on diabetes (T1D, T2D) and its complications (D‐NEP, D‐RET, and D‐NEU) in both discovery and validation phases. 95% CI, 95% confidence interval; D‐NEP, diabetic nephropathy; D‐NEU, diabetic neuropathy; D‐RET, diabetic retinopathy; GWAS, genome‐wide association study; IVW, inverse‐variance weighted; MR, Mendelian randomization; OR, odds ratio; RLS, restless legs syndrome; SNPs, single‐nucleotide polymorphisms; T1D, Type 1 diabetes; T2D, Type 2 diabetes.

Heritability for RLS, T1D, T2D, D‐NEP, D‐RET, and D‐NEU was assessed for both the discovery and validation studies using LDSC regression analysis. The LDSC results revealed significant heritability in 9 out of 11 phenotypes analyzed, including RLS, T1D, T2D, D‐NEP, and D‐NEU (GCST90129436), as well as D‐RET (finn‐b‐DM_RETINOPATHY). Common genetic variants explained between 1.9% and 16.0% of phenotypic variance, with T2D (finn‐b‐E4_DM2) showing the highest heritability (*h* ^2^ = 0.160, *p* = 1.28E − 31), followed by RLS (*h* ^2^ = 0.145, *p* = 5.79E − 08). Detailed results are provided in Figure 2A.

### 3.2 Forward MR and Sensitive Analyses

The forward MR analysis investigated the causal relationship between RLS and diabetes mellitus (T1D and T2D), as well as diabetic complications, including D‐NEP, D‐RET, and D‐NEU. The analysis utilized 11 IVs for RLS as the exposure, with *F*‐statistic values ranging from 32.33 to 880.53, effectively mitigating concerns about weak instrument bias (Table S1).

In the discovery phase, the forward MR analysis indicated a genetic causal association between RLS and diabetic nephropathy (estimated ORs: IVW = 1.049, *p* = 0.0238; IVW radial = 1.049, *p* = 0.0238; RAPS = 1.049, *p* = 0.2851), as detailed in Figure 2D and Table 2. The IVW method, which assumes all IVs are valid, is a weighted regression model that regresses the effect sizes of each genetic instrument on exposure and outcome, providing results with high statistical power. IVW radial, on the basis of the original IVW model, refines the analysis of residuals and outliers to better detect and exclude invalid instruments, thus reducing the impact of these outliers. The RAPS method, which is more stringent by incorporating penalty terms to minimize the influence of invalid instruments, such as those with pleiotropy or bias, yielded consistent effect estimates, though no significant association was found (*p* = 0.2851). This indicates potential pleiotropy may have affected the results. Further sensitivity analysis using MR‐Egger indicated intercepts close to zero (*p* = 0.6094), suggesting no horizontal pleiotropy bias (Figure 2B). The consistency in effect estimates across IVW, IVW radial, and RAPS analyses indicated robustness of the findings, despite no significant causal association with other diabetic complications.

**TABLE 2:** Forward Mendelian randomization results with restless legs syndrome as the exposure in both discovery and validation phases.

Phase	Outcomes	SNPs (*n*)	IVW		IVW radial		RAPS	
			OR (95% CI)	*p*	OR (95% CI)	*p*	OR (95% CI)	*p*
Discovery	T1D	11	0.995 (0.967, 1.023)	0.7237	0.995 (0.967, 1.023)	0.7237	0.995 (0.965, 1.027)	0.7568
	T2D	11	1.011 (0.994, 1.029)	0.1947	1.011 (0.994, 1.029)	0.1947	1.013 (0.995, 1.031)	0.1583
	D‐NEP	11	1.049 (1.006, 1.093)	0.0238	1.049 (1.006, 1.093)	0.0238	1.049 (0.961, 1.145)	0.2851
	D‐RET	11	0.843 (0.676, 1.051)	0.1297	0.843 (0.676, 1.051)	0.1297	0.869 (0.714, 1.058)	0.1609
	D‐NEU	11	0.984 (0.851, 1.137)	0.8250	0.984 (0.851, 1.137)	0.8250	0.962 (0.854, 1.085)	0.5286
Validation	T1D	11	1.029 (0.976, 1.084)	0.2907	1.029 (0.976, 1.084)	0.2907	1.028 (0.976, 1.084)	0.2949
	T2D	11	1.023 (0.994, 1.052)	0.1201	1.022 (0.994, 1.052)	0.1202	1.019 (0.995, 1.043)	0.1253
	D‐NEP	11	1.067 (1.023, 1.114)	0.0028	1.067 (1.023, 1.114)	0.0028	1.068 (1.013, 1.125)	0.0140
	D‐RET	11	0.998 (0.977, 1.02)	0.8763	0.998 (0.977, 1.02)	0.8763	0.998 (0.973, 1.025)	0.8957
	D‐NEU	11	1.04 (0.936, 1.156)	0.4691	1.04 (0.936, 1.156)	0.4691	1.037 (0.937, 1.149)	0.4810

Abbreviations: 95% CI, 95% confidence interval; D‐NEP, diabetic nephropathy; D‐NEU, diabetic neuropathy; D‐RET, diabetic retinopathy; IVW, inverse‐variance weighted; RAPS, robust adjusted profile score; SNPs, single‐nucleotide polymorphisms; T1D, Type 1 diabetes; T2D, Type 2 diabetes.

In the validation phase using the FinnGen GWAS database, the results were consistent with those of the discovery study, indicating a strong genetic association between RLS and an increased risk of diabetic nephropathy (estimated ORs: IVW = 1.067, *p* = 0.0028; IVW radial = 1.067, *p* = 0.0028; RAPS = 1.068, *p* = 0.0140) (Figure 2C,D; Table 2). All three methods produced significant results, further confirming the robustness of the findings. No significant causal relationships were found for other forms of diabetes or diabetic complications. Thus, both the discovery and validation studies concluded that RLS is a risk factor for diabetic nephropathy. More detailed results are presented in Table S2.

In the validation phase using data from the FinnGen GWAS database, results were consistent with those of the discovery study, indicating a strong genetic association between RLS and an increased risk of diabetic nephropathy (estimated ORs: IVW = 1.067, *p* = 0.0028; IVW radial = 1.067, *p* = 0.0028; RAPS = 1.068, *p* = 0.0140) (Figure 2C,D; Table 2). Each method yielded significant results, further supporting the robustness of these findings. No significant genetic associations were observed for other diabetes types or diabetic complications. Consequently, both the discovery and validation phases suggest that RLS predisposes individuals to diabetic nephropathy. Detailed results are provided in Table S2.

**FIGURE 3:**
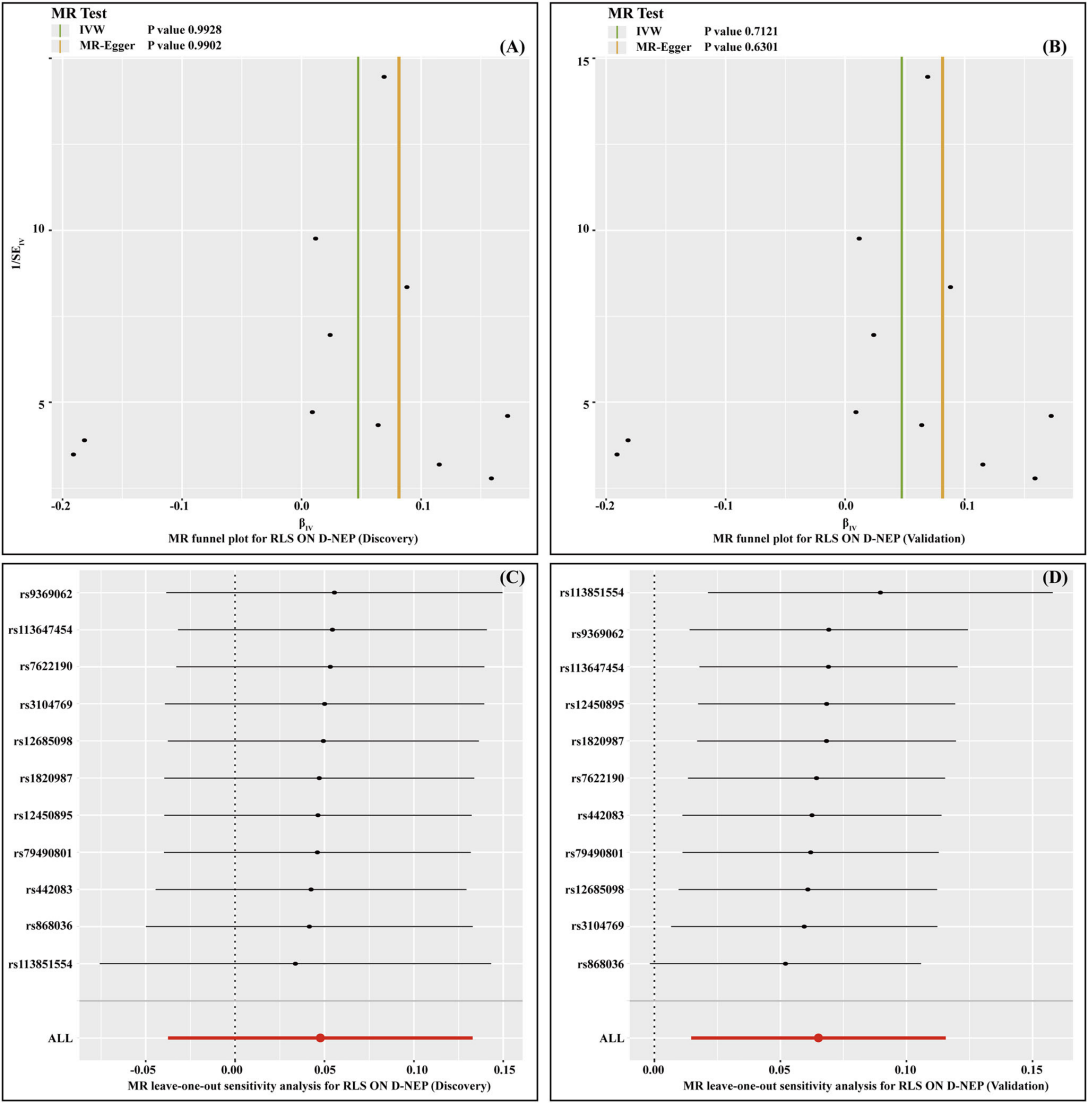
Sensitivity analyses for the causal effect of RLS on D‐NEP using funnel plots and leave‐one‐out analyses. (A) Funnel plot for MR analysis of RLS on D‐NEP during the discovery phase; (B) funnel plot for MR analysis of RLS on D‐NEP during the validation phase; (C) leave‐one‐out sensitivity analysis for RLS on D‐NEP in the discovery phase; (D) leave‐one‐out sensitivity analysis for RLS on D‐NEP in the validation phase. D‐NEP, diabetic nephropathy; IVW, inverse‐variance weighted; MR, Mendelian randomization; RLS, restless legs syndrome.

In sensitivity analyses for exposures and outcomes demonstrating significant causal associations, Cochran's *Q*‐test detected no heterogeneity in either the IVW or MR‐Egger models for the specific exposure‐outcome pairs, with *p* values ranging from 0.6300 to 0.9982 (Table 3, Table S3), as also illustrated in the funnel plots (Figure 3A,B). Furthermore, the MR‐Egger intercept test indicated no evidence of directional pleiotropy, with *p* values between 0.6094 and 0.7937 (Table 3). Additionally, the leave‐one‐out analysis confirmed that the overall IVW estimates were not influenced by any single SNP, supporting the robustness of the findings (Figure 3C,D).

**TABLE 3:** Sensitive analysis results for forward Mendelian randomization (MR) study in both discovery and validation phases.

Phase	Outcomes	SNPs (*n*)	Horizontal pleiotropy		Heterogeneity		
			Egger intercept	*p*	*Q*	*p*	*I^2^*
Discovery	T1D	11	0.0002	0.9880	8.64	0.5667	0
	T2D	11	0.0033	0.5709	14.49	0.1516	31.01
	D‐NEP	11	−0.0142	0.6094	2.36	0.9928	0
	D‐RET	11	−0.0751	0.3073	16.89	0.0769	40.79
	D‐NEU	11	−0.0655	0.1626	18.69	0.0444	46.48
Validation	T1D	11	−0.0033	0.8516	16.14	0.0958	38.03
	T2D	11	0.0084	0.3690	18.93	0.0412	47.16
	D‐NEP	11	0.0043	0.7937	7.14	0.7121	0
	D‐RET	11	0.0096	0.2582	7.48	0.6799	0
	D‐NEU	11	0.0327	0.3511	18.84	0.0424	46.91

Abbreviations: D‐NEP, diabetic nephropathy; D‐NEU, diabetic neuropathy; D‐RET, diabetic retinopathy; SNPs, single‐nucleotide polymorphisms; T1D, Type 1 diabetes; T2D, Type 2 diabetes.

### 3.3 Reverse MR and Sensitive Analyses

A reverse MR analysis was performed to investigate whether diabetes (T1D and T2D) and its complications (D‐NEP, D‐RET, and D‐NEU) are genetically linked to a higher likelihood of developing RLS. Appropriate IVs were selected for each exposure. In the discovery study, the qualifying SNPs for T1D, T2D, D‐NEP, D‐RET, and D‐NEU were 89, 183, 1, 1, and 1, respectively, with the lowest *F*‐statistic observed at 29.60 (*F* > 10). For the validation study, the eligible SNPs were 16, 61, 2, 13, and 4, respectively, with the lowest *F*‐statistic observed at 29.73, as shown in Table S4.

**FIGURE 4:**
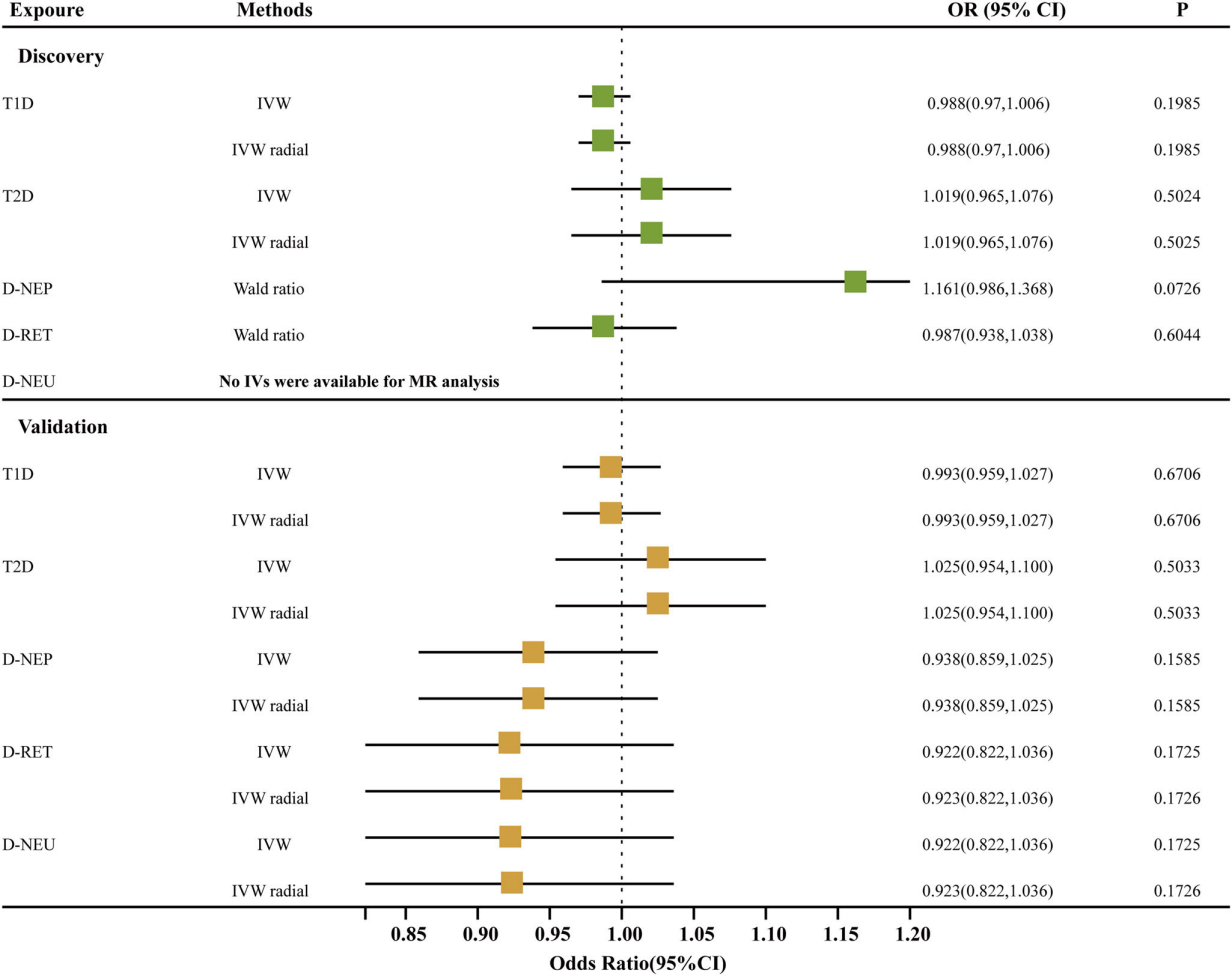
Forest plot presenting causal effect estimates of diabetes (T1D, T2D) and its complications (D‐NEP, D‐RET, and D‐NEU) on RLS in both discovery and validation phases. D‐NEP, diabetic nephropathy; D‐NEU, diabetic neuropathy; D‐RET, diabetic retinopathy; IVW, inverse‐variance weighted; RLS, restless legs syndrome; T1D, Type 1 diabetes; T2D, Type 2 diabetes.

The IVW, IVW radial, and RAPS reverse MR analyses indicated no significant causal relationship between diabetes, its complications, and RLS in both the discovery and validation phases, except for D‐NEU (GCST90129436), for which no suitable IVs were available under stringent criteria (Table 4, Figure 4). More detailed results of the reverse MR analyses can be found in Tables S5 and S6. However, significant heterogeneity or pleiotropy (*p* < 0.05) was detected in T1D and T2D during heterogeneity and horizontal pleiotropy testing (Tables S7 and S8). To ensure robustness, outliers were identified and excluded using the radial IVW and MR‐Egger methods, after which the analyses were repeated. The corrected analyses included T1D datasets (GCST90014023 and finn‐b‐E4_DM1) with adjusted SNP counts of 78 and 14, respectively, and T2D datasets (GCST90018926 and finn‐b‐E4_DM1) with adjusted SNP counts of 158 and 53, respectively. For specific outlier information, refer to Table S9. The revised MR analyses continued to show no significant causal relationship, and no significant heterogeneity or horizontal pleiotropy was detected (Table 5, Tables S10 and S11).

**TABLE 4:** Reverse Mendelian randomization results with Type 1 diabetes (T1D), Type 2 diabetes (T2D), diabetic nephropathy (D‐NEP), diabetic retinopathy (D‐RET), diabetic neuropathy (D‐NEU) as the exposures in both discovery and validation phases.

Phase	Exposures	SNPs (*n*)	IVW/Wald ratio		IVW radial		RAPS	
			OR (95% CI)	*p*	OR (95% CI)	*p*	OR (95% CI)	*p*
Discovery	T1D	78	0.988 (0.97, 1.006)	0.1985	0.988 (0.970, 1.006)	0.1985	0.989 (0.966, 1.012)	0.3472
	T2D	158	1.019 (0.965, 1.076)	0.5024	1.019 (0.965, 1.076)	0.5025	1.018 (0.951, 1.091)	0.6034
	D‐NEP	1	1.161 (0.986, 1.368)	0.0726	NA	NA	1.161 (0.974, 1.385)	0.096
	D‐RET	1	0.987 (0.938, 1.038)	0.6044	NA	NA	0.987 (0.937, 1.039)	0.6139
	D‐NEU	0	NA	NA	NA	NA	NA	NA
Validation	T1D	14	0.993 (0.959, 1.027)	0.6706	0.993 (0.959, 1.027)	0.6706	0.991 (0.953, 1.031)	0.6630
	T2D	53	1.025 (0.954, 1.100)	0.5033	1.025 (0.954, 1.100)	0.5033	1.025 (0.942, 1.115)	0.5731
	D‐NEP	2	0.938 (0.859, 1.025)	0.1585	0.938 (0.859,1.025)	0.1585	0.938 (0.858, 1.026)	0.1642
	D‐RET	11	0.922 (0.822, 1.036)	0.1725	0.923 (0.822, 1.036)	0.1726	0.932 (0.835, 1.041)	0.2109
	D‐NEU	11	0.922 (0.822, 1.036)	0.1725	0.923 (0.822, 1.036)	0.1726	0.932 (0.835, 1.041)	0.2109

*Note*: “NA” indicates that the number of SNPs was insufficient to perform the analysis. When only a single SNP was available, the Wald ratio method was used for analysis.

Abbreviations: 95% CI, 95% confidence interval; IVW, inverse‐variance weighted; RAPS, robust adjusted profile score; SNPs, single‐nucleotide polymorphisms.

**TABLE 5:** Sensitive analysis results for reverse Mendelian randomization (MR) study in both discovery and validation phases.

Phase	Exposures	SNPs (*n*)	Horizontal pleiotropy		Heterogeneity		
			Egger intercept	*p*	*Q*	*p*	*I^2^*
Discovery	T1D	78	−0.0050	0.2360	51.68	0.9882	0
	T2D	158	−0.0082	0.1031	105.56	0.9994	0
	D‐NEP	1	NA	NA	NA	NA	NA
	D‐RET	1	NA	NA	NA	NA	NA
	D‐NEU	0	NA	NA	NA	NA	NA
Validation	T1D	14	0.0232	0.1269	10.55	0.6481	0
	T2D	53	−0.0133	0.1467	40.32	0.8805	0
	D‐NEP	2	NA	NA	1.03	0.3096	3.1195
	D‐RET	11	0.0198	0.2260	13.56	0.1940	26.2509
	D‐NEU	11	0.0074	0.8875	0.61	0.7375	0

*Note*: “NA” indicates that sensitivity analyses could not be performed due to an insufficient number of SNPs.

Abbreviations: D‐NEP, diabetic nephropathy; D‐NEU, diabetic neuropathy; D‐RET, diabetic retinopathy; SNPs, single‐nucleotide polymorphisms; T1D, Type 1 diabetes; T2D, Type 2 diabetes.

## 4 Discussion

This study applied a bidirectional MR framework to examine the potential causal relationship between RLS and diabetes, including its complications—diabetic nephropathy, retinopathy, and neuropathy. Utilizing large‐scale GWAS data, our findings provide evidence supporting a causal effect of RLS on the risk of developing diabetic nephropathy. This association was consistently observed in both the discovery and validation cohorts. Conversely, we did not find evidence of a causal effect of diabetes or its complications on the risk of RLS in our reverse MR analysis.

Our forward MR analysis demonstrated that genetic liability to RLS increases the risk of diabetic nephropathy. The use of multiple MR methods—including IVW, IVW radial, and RAPS—yielded consistent results, reinforcing the robustness of our findings. Sensitivity analyses further supported the validity of the causal relationship, showing no significant heterogeneity or horizontal pleiotropy that could bias the results.

The causal link between RLS and diabetic nephropathy established by our MR analysis is supported by several intersecting biological mechanisms. First, a primary pathway is likely mediated through sleep disruption. RLS is clinically defined by an uncontrollable urge to move the legs, which frequently leads to significant sleep fragmentation and deprivation (Demirtaş and Dolu 2022; Chaiard and Weaver 2019; Diaz et al. 2021; Bonakis et al. 2020). It is well established that chronic poor sleep adversely affects glucose metabolism and insulin sensitivity, which can directly exacerbate diabetic complications (Yi et al. 2023; Kothari et al. 2021; St‐Onge et al. 2023; Schipper et al. 2021). Moreover, large‐scale prospective data have independently linked unhealthy sleep patterns to a decline in overall kidney function (Li et al. 2023).

Beyond these systemic effects, a growing body of evidence suggests that RLS and diabetic nephropathy (DN) may share more direct pathophysiological roots, particularly concerning dopaminergic dysregulation and iron‐dependent cellular pathways (Aini et al. 2024; Morais et al. 2023; Lyu et al. 2020; Kocar et al. 2020).

Dopaminergic axis: The kidney possesses its own intrarenal dopamine system that is critical for modulating glomerular hemodynamics, tubular sodium transport, oxidative stress, and fibrosis. Under diabetic and other oxidative/metabolic stress conditions, this protective system is often impaired; studies show that such stresses can down‐regulate renal D1/D2 receptor expression and disrupt receptor–G‐protein coupling (Yang et al. 2021; Du et al. 2024). This disruption can drive key pathological features of early DN, including sodium retention and glomerular hyperfiltration (Horita et al. 2024). Furthermore, experimental models demonstrate that activation of the D1 receptor protects podocytes from oxidative stress, whereas D2 receptor dysfunction is linked to pro‐inflammatory and pro‐fibrotic phenotypes in renal cells (Shao et al. 2020; Liao et al. 2025). Thus, the systemic dopaminergic dysfunction inherent to RLS could plausibly exacerbate a pre‐existing or developing dopaminergic deficit in the diabetic kidney, thereby accelerating nephropathy progression.

Iron metabolism and ferroptosis: The connection via iron metabolism is similarly compelling. RLS is frequently associated with systemic or brain iron deficiency, and the interplay between iron status and RLS symptoms is well documented. In diabetic kidneys, iron metabolism is also profoundly disturbed (Jin et al. 2024). An emerging consensus indicates that excess labile iron in the kidney catalyzes the production of reactive oxygen species (ROS), triggers extensive lipid peroxidation, and initiates ferroptosis—an iron‐dependent form of regulated cell death—in tubular, glomerular, and mesangial cells (Mu et al. 2025). This process is now increasingly recognized as a key driver of DN progression. Supporting this, recent studies have reported that ferroptosis‐related gene signatures and circulating iron indices correlate with DN severity, whereas pharmacological iron chelators or specific ferroptosis inhibitors show promise in mitigating experimental DN (Chu et al. 2024; Miao et al. 2023).

Collectively, these pathways—chronic sleep disturbance, impaired renal dopaminergic signaling, and iron‐induced ferroptosis—provide substantial biological plausibility for the causal relationship observed in our analysis. They represent interconnected mechanisms through which a genetic liability for RLS could translate into an increased risk for diabetic nephropathy. Our findings, therefore, align with and integrate these intersecting areas of research, strengthening the rationale for monitoring and managing RLS in patients with diabetes.

The absence of a causal effect of diabetes and its complications on RLS in our reverse MR analysis suggests that the relationship may be unidirectional. This has important clinical implications. Recognizing RLS as a risk factor for diabetic nephropathy highlights the need for clinicians to monitor and manage RLS symptoms in patients with diabetes proactively. Early intervention for RLS may help reduce the risk or delay the progression of nephropathy in these patients.

Our study has several strengths, including the use of large, well‐characterized GWAS datasets and the replication of findings across independent cohorts. The rigorous selection of IVs and comprehensive sensitivity analyses enhance the credibility of our results. However, there are limitations to consider. First, our study was restricted to populations of European ancestry. As genetic architecture can vary across ethnicities, this limits the generalizability of our findings to other non‐European groups. Next, the reverse MR analysis was limited by low statistical power. For some diabetic complications, only a few IVs were available after applying stringent selection criteria. This increases the risk of false‐negative results, and thus the null findings for the reverse causal analysis should be interpreted with caution. Third, although MR analysis reduces confounding, it cannot eliminate bias from pleiotropy if genetic variants affect the outcome through pathways other than exposure.

Future studies should focus on validating our results across diverse populations and examining the biological mechanisms linking RLS with diabetic nephropathy. Longitudinal studies examining whether treatment of RLS can reduce the incidence or severity of nephropathy in diabetic patients would be valuable. Additionally, investigating the potential role of shared genetic pathways or environmental influences could shed additional light on the relationship between these conditions.

## 5 Conclusion

This study provides evidence supporting a causal effect of RLS on the risk of diabetic nephropathy. These findings underscore the importance of recognizing and managing RLS in patients with diabetes as a potential strategy to prevent or mitigate renal complications. Integrating RLS assessment into routine diabetes care may have significant implications for patient outcomes.

## Author Contributions

**Yun Lin**: writing – original draft, conceptualization, investigation, methodology, visualization, data curation. **Xiaorui Cai**: writing – original draft, investigation, validation. **Haohao Chen**: writing – review and editing, conceptualization, investigation, data curation, methodology, visualization. **Xiaoling Tang**: writing – review and editing, conceptualization.

## Conflicts of Interest

The authors declare no conflicts of interest.

## Peer Review

The peer review history for this article is available at https://publons.com/publon/10.1002/brb3.70696

## Supporting information




**Supplementary Figure 1**: brb370696‐sup‐0001‐Figure1.docx

**Supplementary Tables**: brb370696‐sup‐0002‐Figure1.jpeg

**Supplementary Tables**: brb370696‐sup‐0002‐Tables.xlsx

## Data Availability

The data are accessible in both the main manuscript and the supplementary materials. The original GWAS data can be retrieved from their respective online databases: https://www.ebi.ac.uk/gwas/ and https://www.finngen.fi/en.
