# Supplementary material for: Genetic Insights Into the Link Between Restless Legs Syndrome and Diabetic Nephropathy Risk
Source: Brain Behav. 2025 Jul 21;15(7):e70696. doi: 10.1002/brb3.70696 (PMC12277665; doi:10.1002/brb3.70696)
Supplement: Supplementary file 1 — Supplementary Figure 1: brb370696‐sup‐0001‐Figure1.docx [file BRB3-15-e70696-s001.docx]

Supplementary Figure Legend

Supplementary Figure 1. Schematic overview of the study design and analytical pipeline.

The figure provides a comprehensive overview of the study, detailing the bidirectional Mendelian randomization (MR) study design, the specific GWAS data sources used for exposures and outcomes, and the complete step-by-step analytical workflow. The workflow includes the criteria for instrumental variable (IV) selection, data harmonization, outlier removal for pleiotropy correction, primary MR analysis methods, and the suite of sensitivity analyses used to validate the findings.

Abbreviations: D-NEP, diabetic nephropathy; D-NEU, diabetic neuropathy; D-RET, diabetic retinopathy; EU-RLS-GENE, international Restless Legs Syndrome Genetics Consortium; GWAS, genome-wide association study; ID, identifier; IVW, inverse-variance weighted; P, P-value; r², R-squared (a measure of linkage disequilibrium); RAPS, robust adjusted profile score; RLS, restless legs syndrome; SNPs, single-nucleotide polymorphisms; T1D, type 1 diabetes; T2D, type 2 diabetes.
