# Supplementary figures and images for: Genetic Insights Into the Link Between Restless Legs Syndrome and Diabetic Nephropathy Risk
Source: Brain Behav. 2025 Jul 21;15(7):e70696. doi: 10.1002/brb3.70696 (PMC12277665; doi:10.1002/brb3.70696)

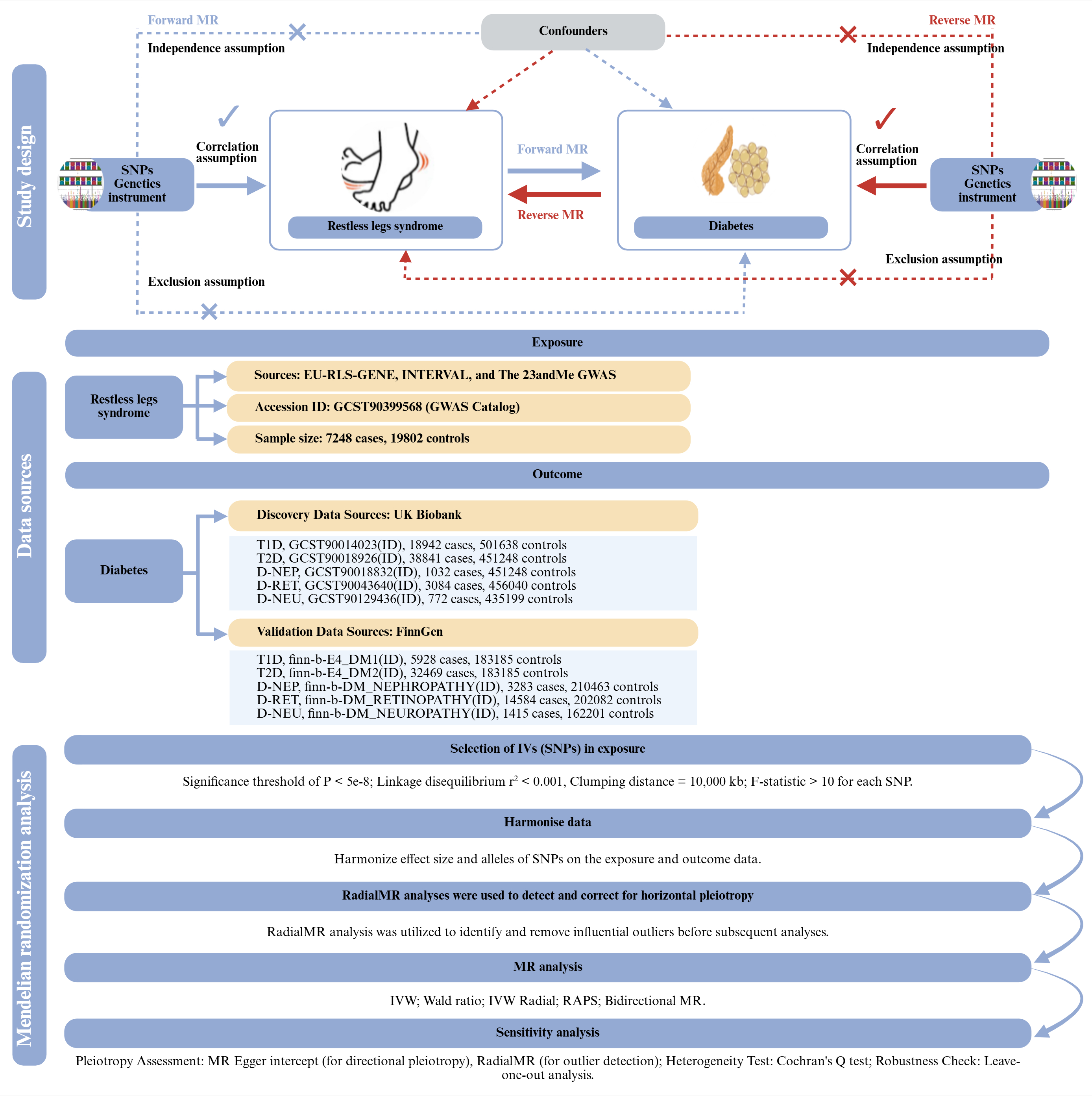

Supplement: Supplementary file 2 — Supplementary Tables: brb370696‐sup‐0002‐Figure1.jpeg [file BRB3-15-e70696-s003.jpeg]
